# Point-of-Care (POC) Urinary L-Type Fatty Acid-Binding Protein (u-LFABP) Use in Critically Ill, Very Preterm Neonates

**DOI:** 10.1155/2022/4684674

**Published:** 2022-03-19

**Authors:** Henny Adriani Puspitasari, Eka Laksmi Hidayati, Retno Palupi-Baroto, Diashati Ramadhani Mardiasmo, Rosalina Dewi Roeslani

**Affiliations:** ^1^Division of Nephrology, Department of Child Health, Faculty of Medicine, Universitas Indonesia, Jakarta, Indonesia; ^2^Division of Nephrology, Department of Child Health, Faculty of Medicine, Public Health and Nursing, Universitas Gadjah Mada, Yogyakarta, Indonesia; ^3^Medical Technology Cluster, Indonesia Medical Education and Research Institute, Faculty of Medicine, Universitas Indonesia, Jakarta, Indonesia; ^4^Division of Perinatology, Department of Child Health, Faculty of Medicine, Universitas Indonesia, Jakarta, Indonesia

## Abstract

Preterm neonates are born with fewer functional nephrons, rendering them vulnerable to secondary insult. These insults are associated with acute kidney injury (AKI); thus, structural damage must be detected as early as possible. Urinary L-type fatty acid-binding protein (u-LFABP) has been proposed as a highly suitable kidney injury biomarker during prematurity. We aimed to analyze the use of POC u-LFABP in critically ill, very preterm neonates. This study was conducted at the neonatal intensive care unit (NICU), Dr. Cipto Mangunkusumo General Hospital, from November to December 2020. Baseline characteristics were recorded from electronic medical records. u-LFABP examination utilized stored urine samples from a previous study and was performed using a LFABP POC test kit. The proportion of abnormal u-LFABP (83.3%) was highest at 72 hours. Neonates with older gestational age (0–48 hours; *p*=0.017) and higher birth weight (0–48 hours; *p*=0.022, 72 hours; *p*=0.013) had normal u-LFABP levels. Neonates exposed to nephrotoxic agents showed higher proportion of abnormal u-LFABP (0–48 hours; *p*=0.006). Longer invasive mechanical ventilation (IMV) period was observed in neonates with abnormal u-LFABP levels at 0–48 hours (7.44 ± 7.9 vs. 1.50 ± 2.9 days; *p*=0.011). We found an association between complication rates and poorer disease outcome trends with abnormal u-LFABP; however, this relationship was not supported statistically. In conclusion, this study demonstrated that u-LFABP can be detected using bedside POC kit in critically ill very preterm neonates and those exposed to nephrotoxic agents may be at risk for kidney injury, confirmed by abnormal u-LFABP levels.

## 1. Introduction

The WHO defines preterm birth as all births before 37 weeks of gestation, with subcategories including very (<32 weeks' gestation) and extreme (<28 weeks' gestation) preterm births [1]. In 2014, Indonesia was stated as the fifth leading country regarding high numbers of preterm births, estimated at 10.4% of all live births [2]. In 2018, our hospital, a tertiary national referral facility, observed 507 preterm live births, of which 112 were very preterm. The survival rate was reportedly 58.9% [3]. One of the preterm birth complications that is a leading cause of death is acute kidney injury (AKI), which is estimated to occur in 30% of preterm neonates and around 18% in neonates born at 29 weeks to less than 36 weeks of gestational age [4].

Intrauterine nephrogenesis begins at 8 weeks of gestation and continues up to approximately 34–36 weeks of gestation, with over 60% of nephrons forming within the last pregnancy trimester [5]. Nephrogenesis may continue up to 40 days postnatal in preterm neonates; however, they do not achieve full nephrogenesis [6]. Hence, such neonates are born with fewer functional nephrons, have immature tubules, abnormal glomeruli morphology, and a higher glomerular volume (suggestive of hyperfiltration), thereby limiting kidney function [7, 8]. Such impairments render preterm neonates vulnerable to secondary injuries during intensive care, primarily caused by hypoperfusion (blood loss, asphyxia, patent ductus arteriosus (PDA), or sepsis) and nephrotoxic insult (due to diuretics, nonsteroidal anti-inflammatory drugs (NSAIDs), antifungals, and antibiotics). [5] These events lead to AKI, which correlates with a longer hospital stay and higher mortality rate and in later life increases the risk of chronic kidney disease (CKD) and hypertension [9].

Serum creatinine and urine output are functional markers of AKI that have been used despite their late rising levels in the disease process. Significant scientific efforts have focused on the discovery of biomarkers for detecting early injury in AKI events, particularly in preterm neonates, to improve outcomes. Urinary L-type fatty acid-binding protein (u-LFABP) is localized in proximal tubules of the human kidneys and excreted into urine during hypoxic stress, independently from serum LFABP levels. Therefore, u-LFABP can predict tubular cell injury examined by histological analysis before the rise of serum creatinine. A study by Portilla et al. demonstrated a significantly increased u-LFABP within the first 4 hours of cardiac surgery in patients who later developed AKI, preceding the rise of serum creatinine [10]. In neonates, u-LFABP was shown to predict AKI at 0 and 2 hours post cardiac surgery, with areas under the curve (AUCs) of 0.84 and 0.89, respectively [11]. Additionally, preterm neonates excreted markedly more u-LFABP than healthy term neonates on days 25–30 [12]. Thus, u-LFABP may have significant potential for predicting AKI when used at bedside point-of-care (POC) in very preterm neonates. The first objective of this study was to describe the pattern of u-LFABP levels in critically ill, very preterm neonates. The second objective was to determine the association of u-LFABP and clinical factors as well as disease outcome in these neonates.

## 2. Materials and Methods

### 2.1. Study Design, Setting, and Population

This study was a retrospective cohort study conducted at the neonatal intensive care unit (NICU), Cipto Mangunkusumo General Hospital from November to December 2020. Participants were very preterm neonates born between 28 and 32 weeks of gestational age, recruited consecutively for a previous study entitled “Does High Protein Intake Cause Tubular Injury in Very Preterm Neonates?” [13]. Exclusion criteria included any major congenital abnormalities (e.g., complex congenital heart disease, congenital anomalies of the kidney and urinary tract (CAKUT), craniofacial and cerebral anomalies, and congenital abdominal anomalies), intrauterine growth restriction (IUGR), nephrotoxic agent exposure during pregnancy, urine collection difficulties, and consent refusal. Patients were followed up from birth until day 21, unless deceased.

### 2.2. Urine Collection and Storage

Frozen urine samples from the previous study [13] were thawed for u-LFABP examination. Those frozen urine samples were previously collected at three different time points: 0–48 hours, 72 hours, and 21 days using a urine collector or urethral catheter. Samples were kept in a refrigerator at 13–15°C for a maximum of 12 hours and sent to a standardized laboratory in a cooler box at that same temperature. Samples were centrifuged, and supernatants were frozen at −70°C and stored for a maximum of 3 months.

### 2.3. Data Collection

Data were recorded from electronic medical records (EMRs) and included sex, gestational age, anthropometry (birth weight, birth length, and head circumference), perinatal events (i.e., PDA, necrotizing enterocolitis (NEC), and sepsis), administration of vasopressor medication and nephrotoxic agents during hospitalization, periventricular-intraventricular hemorrhage (PIVH), the use of invasive mechanical ventilation (IMV), length of stay, and death. All perinatal events were diagnosed by a neonatologist.

### 2.4. u-LFABP Examination and Definition

u-LFABP was carried out using an LFABP POC test kit (CMIC, Tokyo, Japan). One hundred *μ*L of urine sample was added to a pretreatment microtube, mixed with pretreatment reagent, and added into the test cassette. After 15 minutes, the color intensity of the test line was compared with the reference card provided, and the results were interpreted as either negative (u-LFABP <12.5 ng/mL), positive 1 (u-LFABP ≥12.5 ng/mL and <100 ng/mL), or positive 2 (u-LFABP ≥100 ng/mL). Normal results were equivalent to negative results, whereas abnormal results were those with ≥12.5 ng/mL (positive 1 and 2).

### 2.5. Other Variables

PDA was diagnosed based on echocardiography indicating hemodynamically significant left-to-right shunt, leading to pulmonary overcirculation and systemic hypoperfusion [14]. NEC was defined according to modified Bell's staging [15]. Sepsis was diagnosed based on maternal (fever during pregnancy, rupture of membranes, chorioamnionitis, foul-smelling discharge, urinary tract infection, and sexually transmitted disease) and neonatal risk factors (low birthweight and prematurity) as well as hypotension, tachycardia or bradycardia, temperature >37.7 or <35.5°C, respiratory distress, leucocyte count >12,000/*µ*L or <4,000/*µ*L, high CRP, and high I/T ratio regardless blood culture result [16]. Vasopressor exposure was defined as exposure to dopamine, dobutamine, epinephrine, and/or norepinephrine. Nephrotoxic exposure was defined as exposure to nephrotoxic agents listed in the “Baby NINJA” study [17]. PIVH was diagnosed based on head ultrasonography (USG) which was performed by a pediatric radiologist at the earliest at 5 days of age. IMV use was defined as use of mechanical ventilator to deliver positive pressure via an endotracheal tube into the lungs.

### 2.6. Statistical Analysis

All statistical analyses were performed using IBM SPSS Statistics for Macintosh, version 21.0, released in 2012 (Armonk, NY; IBM Corp). The data are presented as a percentage for categorical variables, mean ± standard deviation (SD) for normally distributed data, and median (minimal–maximum) for non-normally distributed data. Bivariate analysis between both numerical datasets was performed with an unpaired *t*-test and a Mann–Whitney test for normally and non-normally distributed data, respectively. Bivariate analysis between categorical and numerical data was carried out with Fisher's exact test. The normality test was conducted with Shapiro–Wilk test.

### 2.7. Ethics Statement

This research was approved by the Faculty of Medicine of Universitas Indonesia Ethics Committee (KET-85/UN2.F1/ETIK/PPM.00.02/2021). The research was conducted in ethical accordance with the World Medical Association Declaration of Helsinki.

## 3. Results

### 3.1. Patients' Characteristics

**Figure 1:**
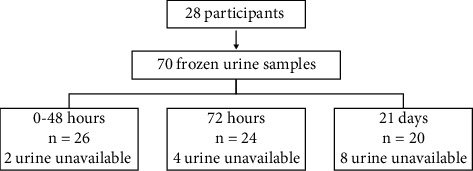
Study population. A total of 70 frozen urine samples from 28 very preterm neonates were available. Of the 70 urine samples, 26 samples were collected at 0–48 hours, 24 samples were collected at 72 hours, and 20 samples were collected at 21 days.

During this study period, 70 frozen urine samples from 28 very preterm neonates were available (Figure 1). Patients were born between 28 and 32 weeks of gestational age, with a mean birth weight of 1277.8 ± 237.4 g and mean birth length of 36.9 ± 2.7 cm (Table 1). Twenty-four neonates were indicative for cardiology consultation and echocardiography examination. We were unable to obtain head ultrasonography results from four patients. Thirteen neonates had PDA, 6/13 (46.3%) resolved spontaneously, 2/13 (15.4%) resolved after 1 cycle of intravenous paracetamol (IV PCT) and 5/13 (38.5%) resolved after IV PCT 2–3 cycle. There were no neonates who required PDA ligation. Of 10 neonates with NEC, 2/10 (20%) had NEC grade 1 and 8/10 (80%) had NEC grade 2. A total of 20 neonates had sepsis, 6/20 (30.0%) were nonsevere and 14/20 (70.0%) were severe. Severe sepsis was defined as sepsis with a leukocyte count <9000/*µ*L and/or CRP >5 ng/mL. Of 28 neonates, 25 (89.3%) were administered nephrotoxic agents, gentamycin only (5/25; 20.0%), amikacin only (5/25; 20.0%), or both gentamycin and amikacin (15/25; 60.0%). There were no other nephrotoxic agents administered during this study.

### 3.2. Pattern of u-LFABP in Critically Ill, Very Preterm Neonates

**Figure 2:**
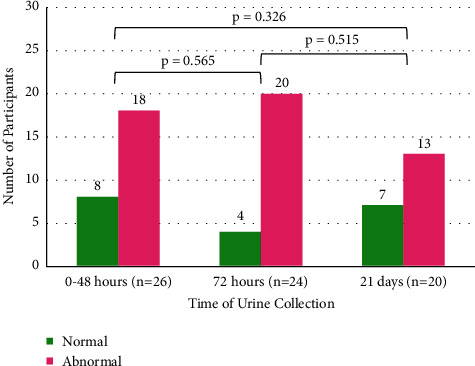
u-LFABP profile across time. Number of abnormal u-LFABP participants was highest at 72 hours. There was no significant difference between u-LFABP levels at each time point (Fisher's exact test).

The proportion of abnormal u-LFABP results at 0–48 hours of age was 18/26 (64.3%), at 72 hours was 20/24 (83.3%), and at 21 days was 13/20 (65.0%). The proportion of abnormal u-LFABP results was found to be highest at 72 hours of age (Figure 2).

### 3.3. u-LFABP and Perinatal Factors and Insults

We found that neonates with higher gestational age (72 hours; *p*=0.017) and higher birth weight (0–48 hours; *p*=0.022, 72 hours; *p*=0.013) had normal u-LFABP levels. Neonates who had PDA and developed NEC and sepsis during hospitalization had a higher proportion of abnormal u-LFABP; however, this association was not supported statistically. In addition, neonates who were exposed to vasopressors and nephrotoxic agents (0–48; *p*=0.022) showed an increased proportion of abnormal u-LFABP (Table 2).

### 3.4. u-LFABP and Disease Severity

We also analyzed u-LFABP levels based on severity level of PDA and NEC. At 21 days, all 3/3 neonates with spontaneously resolved PDA had normal u-LFABP levels while 1/1 and 5/5 neonates who received IV PCT 1 cycle and IV PCT 2-3 cycles all had abnormal u-LFABP levels, respectively (*p*=0.011). We observed neonates with NEC grade 2 had higher proportion of abnormal u-LFABP at all three time points (Table 3).

### 3.5. u-LFABP and Patients' Outcome

To assess disease outcome, we examined the u-LFABP results within the first 72 hours. In general, 18/24 (75%) of our neonates survived during this study. Poor survival, PIVH incidence, and IMV treatment were found more in neonates with abnormal u-LFABP levels, although this association was not statistically significant. Longer IMV periods were noted in neonates with abnormal u-LFABP levels at 0–48 (7.44 ± 7.9 vs. 1.50 ± 2.9 days; *p*=0.011) and 72 (5.40 ± 7.2 vs 4.25 ± 4.9 days; *p*=0.765) hours. Moreover, there was no association between u-LFABP level and hospitalization duration (Table 4).

## 4. Discussion

Our study showed that u-LFABP can be detected in very preterm neonates at the age of 0–48 hours, 72 hours, and 21 days utilizing an LFABP POC kit. The use of such a kit is a practical way to perform a bedside assessment of AKI. This kit measures normal u-LFABP values with cutoff points below 12.5 ng/mL (8.4 ug/gCr). The cutoff value was developed from a study involving healthy adults. We detected a high proportion of neonates with abnormal u-LFABP levels (≥12.5 ng/mL) across all time points during our study (Figure 1). This result was in line with previous studies that showed u-LFABP levels in critically ill, very preterm neonates were higher compared to healthy preterm neonates, healthy term neonates, and healthy adults [12, 18]. The older the age and the greater the birth weight, the higher the proportion of normal u-LFABP was found in our participants (Table 2). These findings support previous studies reporting that proximal tubular maturation is related to gestational age and birth weight [12, 19].

Hypoxia is one factor that induced excretion of LFABP. During kidney ischemia reperfusion injury, LFABP gene responds to hypoxic stress. Therefore, urinary excretion of LFABP mirrors tubular epithelial cells as well as its correlation with ischemic tubular injury severity [20]. All our participants were appropriate for their gestational age (i.e., none of them had IUGR), which eliminates chronic hypoxic events during pregnancy. However, during hospitalization, there were ongoing hypoxic stress events such as asphyxia, PDA, sepsis, respiratory distress syndrome, and vasopressor administration. In our study, neonates with PDA, NEC, sepsis, and vasopressor administration showed a higher proportion of abnormal u-LFABP, although this association was not statistically significant (Table 2). Hypoxic ischemia was reported as one of the risk factors in NEC pathogenesis. During enterocyte death, LFABP enters the circulation and consequently benefits as a marker of intestinal damage. LFABP is raised in patients with NEC and can also be elevated in patients with sepsis and after abdominal surgery or trauma [21].

Our findings also highlighted the higher proportion of abnormal u-LFABP found in very preterm neonates exposed to nephrotoxic agents. Gentamicin and amikacin were antibiotics used in combination with other wide-spectrum antibiotics as first- and second-line treatments for neonatal sepsis in our NICU. These antibiotics produce glomerulotubular injury by impairing segment S1 and S2 of proximal tubules [22]. The “baby NINJA” study showed positive correlations between increased risk of AKI and the number of nephrotoxic agents given during NICU stay and in very preterm neonates [17].

A higher proportion of neonates with abnormal u-LFABP received IMV treatment in our study, and all participants with PIVH had abnormal u-LFABP during the first 72 hours of age. u-LFABP is increasingly excreted during hypoxic events, reflected by the requirement for IMV and PIVH incidence. PIVH is a well-known consequence in critically ill preterm neonates and is significantly associated with cerebral hypoxia. Ferguson et al. concluded that u-LFABP could not be used as a predictor for hospitalization mortality [23]. However, we found in our study that all nonsurvivor's u-LFABP levels were abnormal during the first 72 hours of age. This finding supports previous meta-analysis result showing u-LFABP sensitivity and specificity of 93.2% (95% CI, 66.2–99.0%) and 78.8% (95% CI, 27.0–97.4%) for in-hospital mortality, respectively [24]. u-LFABP was not associated with dialysis treatment or longer hospitalization within 21 days of follow-up in our study. The estimated sensitivity of urinary LFABP level for predicting dialysis requirement was 69.1% (95% CI, 34.6–90.5%), and the specificity was 42.7% (95% CI, 3.1–94.5%) [24]; however, there were no participants requiring dialysis treatment in this study. Various confounding factors may contribute to disease outcome that were not recorded in this research.

The performance of u-LFABP has shown promise in numerous clinical conditions. Elnaldy et al. reported a significant positive correlation between u-LFABP and serum creatinine after 48 hours [25]. Another study also showed significant positive correlations between u-LFABP and %serum creatinine change at 2 hours (u-LFABP median 380 *µ*g/L), 6 hours (u-LFABP median 300 *µ*g/L), and 24 hours (u-LFABP median 160 *µ*g/L) post-cardiac surgery [11]. However, we were unable to show a direct correlation between u-LFABP based on the POC kit result and AKI events due to lack of participants' complete serum creatinine and urine output data.

This is the first study utilizing an LFABP POC kit to detect u-LFABP in critically ill, very preterm neonates. However, our findings were limited by the relatively small sample size, the nature of the retrospective study design, and the lack of available serum creatinine data.

## 5. Conclusion

The findings of this study demonstrate that u-LFABP can be detected with a bedside POC kit in critically ill, very preterm neonates. Normal u-LFABP levels were associated with higher gestational age and higher birth weight. Neonates exposed to nephrotoxic agents may be at risk for kidney injury, confirmed by abnormal u-LFABP levels. Future diagnostic studies should compare u-LFABP with serum creatinine and urine output for early AKI diagnosis as well as determine the u-LFABP normal reference range in critically ill, very preterm neonates.

## Figures and Tables

**Table 1:** Baseline characteristics.

Characteristics	Total (*N* = 28)
Sex^1^	
Male	16 (57.1)
Female	12 (42.9)
Gestational age (weeks)^2^	30 (28–32)
Birth weight (g)^3^	1277.8 (237.4)
Birth length (cm)^3^	36.9 (2.7)
Length of stay (days)^2^	37 (26–79)
Necrotizing enterocolitis^1,4^	9 (32.1)
Patent ductus arteriosus^1^	11 (39.3)
Sepsis^1^	18 (64.3)
Intraventricular haemorrhage^1,4^	6 (25.0)
Nephrotoxic agent^1^	23 (82.1)

^1^ *n* (%) is presented. ^2^Median (min–max) is presented. ^3^Mean (SD) is presented. ^4^*n* = 24.

**Table 2:** Perinatal factors and insults associated with u-LFABP profiles.

	u-LFABP		*p* value
	Abnormal	Normal	
*GA (weeks)* ^1^			
0–48 hours	29 (28–31)	30 (29–32)	0.071
72 hours^+^	29 (28–32)	31 (30–32)	0.017^*∗*^
21 days^+^	30 (28–31)	30 (28–32)	0.837

*Birth weight (g)* ^2^			
0–48 hours	1220 (241.7)	1449.25 (159.8)	0.022^*∗*^
72 hours^+^	1234.70 (203.3)	1548.75 (259.9)	0.013^*∗*^
21 days^+^	1317.69 (259.2)	1261.29 (188.0)	0.619

*PDA* ^3^			
0–48 hours	10/11 (90.9)	1/11 (9.1)	0.149
72 hours^+^	8/10 (80.0)	2/10 (20.0)	1.000
21 days^+^	6/9 (66.7)	3/9 (33.3)	1.000

*NEC* ^3^			
0–48 hours	7/9 (77.8)	2/9 (22.2)	0.667
72 hours^+^	7/8 (87.5)	1/8 (12.5)	1.000
21 days^+^	4/6 (66.7)	2/6 (33.3)	1.000

*Sepsis* ^3^			
0–48 hours	15/18 (83.4)	3/18 (16.7)	0.291
72 hours^+^	13/17 (76.5)	4/17 (23.5)	0.546
21 days^+^	8/12 (66.7)	4/12 (33.3)	1.000

*Vasopressor* ^3^			
0–48 hours	4/4 (100.0)	0/4 (0.0)	0.277
72 hours^+^	3/3 (100.0)	0/3 (0.0)	1.000
21 days^+^	2/3 (66.7)	1/3 (33.3)	0.730

*Nephrotoxic* ^3^			
0–48 hours	18/23 (78.3)	5/23 (21.7)	0.022^*∗*^
72 hours^+^	17/21 (81.0)	4/21 (19.0)	1.000
21 days^+^	11/17 (64.7)	6/17 (35.5)	1.000

^*∗*^ *p* value <0.05; ^+^Missing urine samples; ^1^Median (min–max) is presented, Mann–Whitney; ^2^Mean (SD) is presented, unpaired *t*-test; ^3^*n* (%) is presented, fisher's exact test. GA: gestational age; PDA: patent ductus arteriosus; NEC: necrotizing enterocolitis.

**Table 3:** u-LFABP levels based on disease severity.

	u-LFABP		*p* value
	Abnormal	Normal	
*PDA*			
0–48 hours			0.517
Spontaneous	4/5 (80.0)	1/5 (20.0)	
PCT 1 cycle	2/2 (100.0)	0/2 (0.0)	
PCT >1 cycle	4/4 (100.0)	0/4 (0.0)	
72 hours^+^			0.732
Spontaneous	3/4 (75.0)	1/4 (25.0)	
PCT 1 cycle	2/2 (100)	0/2 (0.0)	
PCT >1 cycle	3/4 (75.0)	1/4 (25.0)	
21 days^+^			0.011^*∗*^
Spontaneous	0/3 (0.0)	3/3 (100.0)	
PCT 1 cycle	1/1 (100.0)	0/1 (0.0)	
PCT >1 cycle	5/5 (100.0)	0/5 (0.0)	

*NEC*			
0–48 hours			1.000
Grade 1	1/1 (100.0)	0/1 (0.0)	
Grade 2	6/8 (75.0)	2/8 (25.0)	
72 hours^+^			1.000
Grade 1	1/1 (100.0)	0/1 (0.0)	
Grade 2	6/7 (85.7)	1/7 (14.3)	
21 days^+^			1.000
Grade 1	1/1 (100.0)	0/1 (0.0)	
Grade 2	3/5 (60.0)	2/5 (40.0)	

^*∗*^ *p* value <0.05. ^+^Missing urine samples. *n* (%) is presented; fisher's exact test. PDA: patent ductus arteriosus; NEC: necrotizing enterocolitis.

**Table 4:** Disease outcome associated with u-LFABP profiles.

	u-LFABP		*p* value
	Abnormal	Normal	
*Death* ^1^			
0–48 hours	6/6 (100.0)	0/6 (0.0)	0.272
72 hours^+^	5/5 (100.0)	0/5 (0.0)	0.530

*IVH* ^1^			
0–48 hours^+^	5/5 (100.0)	0/5 (0.0)	0.135
72 hours	6/6 (100.0)	0/6 (0.0)	0.526

*IMV* ^1^			
0–48 hours	10/12 (83.3)	2/12 (16.7)	0.216
72 hours^+^	9/11 (81.8)	2/11 (18.2)	1.000

*LOS (days)* ^2^			
0–48 hours	35 (26–70)	37 (27–79)	0.911
72 hours^+^	41 (26–79)	34 (30–37)	0.277

^+^Missing urine samples; ^1^*n* (%) is presented, Fisher's exact test; ^2^Median (min–max) is presented, Mann–Whitney. IVH: intraventricular hemorrhage; IMV: invasive mechanical ventilation; LOS: length of stay.

## Data Availability

The datasets used and analyzed during the current study are available from the corresponding author.

## References

[1] Barfield W. D. (2018). Public health implications of very preterm birth. *Clinics in Perinatology*.

[2] Chawanpaiboon S., Vogel J. P., Moller A.-B. (2019). Global, regional, and national estimates of levels of preterm birth in 2014: a systematic review and modelling analysis. *Lancet Global Health*.

[3] Divisi Perinatologi Departemen Ilmu Kesehatan Anak FKUI RSCM (2018). *Profil Data Perinatologi Rumah Sakit Cipto Mangunkusumo*.

[4] Jetton J. G., Boohaker L. J., Sethi S. K. (2017). Incidence and outcomes of neonatal acute kidney injury (AWAKEN): a multicentre, multinational, observational cohort study. *The Lancet Child & Adolescent Health*.

[5] Stritzke A., Thomas S., Amin H., Fusch C., Lodha A. (2017). Renal consequences of preterm birth. *Molecular and cellular pediatrics*.

[6] Galu S. C., Hascoet J. M., Vieux R. (2015). Impact of neonatal factors and nutrition on kidney size in 5-year-old preterm-born children. *American Journal of Perinatology*.

[7] Martinerie L., Pussard E., Yousef N. (2015). Aldosterone-signaling defect exacerbates sodium wasting in very preterm neonates: the premaldo study. *Journal of Clinical Endocrinology & Metabolism*.

[8] De Curtis M., Senterre J., Rigo J. (1990). Renal solute load in preterm infants. *Archives of Disease in Childhood*.

[9] Luyckx V. A., Bertram J. F., Brenner B. M. (2013). Effect of fetal and child health on kidney development and long-term risk of hypertension and kidney disease. *The Lancet*.

[10] Portilla D., Dent C., Sugaya T. (2008). Liver fatty acid-binding protein as a biomarker of acute kidney injury after cardiac surgery. *Kidney International*.

[11] Peco-Antić A., Ivanišević I., Vulićević I. (2013). Biomarkers of acute kidney injury in pediatric cardiac surgery. *Clinical Biochemistry*.

[12] Tsukahara H., Sugaya T., Hayakawa K. (2005). Quantification of L-type fatty acid binding protein in the urine of preterm neonates. *Early Human Development*.

[13] Puspitasari H. A., Trihono P. P., Wahidiyat P. A. (2021). Does high protein intake cause tubular injury in very preterm neonates?. *Research Square*.

[14] Smith A., El-Khuffash A. F. (2020). Defining “haemodynamic significance” of the patent ductus arteriosus: do we have all the answers?. *Neonatology*.

[15] Patel R. M., Ferguson J., McElroy S. J., Khashu M., Caplan M. S. (2020). Defining necrotizing enterocolitis: current difficulties and future opportunities. *Pediatric Research*.

[16] Helguera-Repetto A. C., Soto-Ramírez M. D., Villavicencio-Carrisoza O. (2020). Neonatal sepsis diagnosis decision-making based on artificial neural networks. *Front Pediatr*.

[17] Stoops C., Stone S., Evans E. (2019). Baby NINJA (nephrotoxic injury negated by just-in-time action): reduction of nephrotoxic medication-associated acute kidney injury in the neonatal intensive care unit. *The Journal of Pediatrics*.

[18] Sato R., Suzuki Y., Takahashi G., Kojika M., Inoue Y., Endo S. (2015). A newly developed kit for the measurement of urinary liver-type fatty acid-binding protein as a biomarker for acute kidney injury in patients with critical care. *Journal of Infection and Chemotherapy*.

[19] Awad H., El-Barbary M., Imam S., El-Safty I. (2002). Evaluation of renal glomerular and tubular functional and structural integrity in neonates. *The American Journal of the Medical Sciences*.

[20] Cho E., Yang H. N., Jo S.-K., Cho W.-Y., Kim H.-K. (2013). The role of urinary liver-type fatty acid-binding protein in critically ill patients. *Journal of Korean Medical Science*.

[21] Coufal S., Kokesova A., Tlaskalova-Hogenova H. (2020). Urinary I-fABP, L-FABP, TFF-3, and SAA can diagnose and predict the disease course in necrotizing enterocolitis at the early stage of disease. *Journal of Immunology Research*.

[22] Murphy H. J., Thomas B., Van Wyk B., Tierney S. B., Selewski D. T., Jetton J. G. (2020). Nephrotoxic medications and acute kidney injury risk factors in the neonatal intensive care unit: clinical challenges for neonatologists and nephrologists. *Pediatric Nephrology*.

[23] Ferguson M. A., Vaidya V. S., Waikar S. S. (2010). Urinary liver-type fatty acid-binding protein predicts adverse outcomes in acute kidney injury. *Kidney International*.

[24] Susantitaphong P., Siribamrungwong M., Doi K., Noiri E., Terrin N., Jaber B. L. (2013). Performance of urinary liver-type fatty acid-binding protein in acute kidney injury: a meta-analysis. *American Journal of Kidney Diseases*.

[25] Elnady H. G., Abdalmoneam N., Abu Shady M. M., Hassanain A. I., Ibraheim R. A. M., Abdel Raouf H. (2014). Urinary liver-type fatty acid-binding protein for early detection of acute kidney injury in neonatal sepsis. *Medical Research Journal*.

